# Expression Patterns and Molecular Mechanisms Regulating Drought Tolerance of Soybean [*Glycine max* (L.) Merr.] Conferred by Transcription Factor Gene *GmNAC19*

**DOI:** 10.3390/ijms25042396

**Published:** 2024-02-18

**Authors:** Xiyan Cui, Minghao Tang, Lei Li, Jiageng Chang, Xiaoqin Yang, Hongli Chang, Jiayu Zhou, Miao Liu, Yan Wang, Ying Zhou, Fengjie Sun, Zhanyu Chen

**Affiliations:** 1 College of Life Sciences, Jilin Agricultural University, Changchun 130118, China; cuixiyan@jlau.edu.cn (X.C.); wangyansk@jlau.edu.cn (Y.W.); yzhou@jlau.edu.cn (Y.Z.); 2 Shaanxi Key Laboratory for Animal Conservation, School of Life Sciences, Northwest University, Xi’an 710069, China; 3 Department of Biological Sciences, School of Science and Technology, Georgia Gwinnett College, Lawrenceville, GA 30043, USA; 4 College of Agronomy, Jilin Agricultural University, Changchun 130118, China

**Keywords:** soybean, drought stress and tolerance, NAC transcription factor, proline, malondialdehyde, superoxide dismutase, peroxidase, catalase, soluble protein, soluble sugar

## Abstract

NAC transcription factors are commonly involved in the plant response to drought stress. A transcriptome analysis of root samples of the soybean variety ‘Jiyu47’ under drought stress revealed the evidently up-regulated expression of *GmNAC19*, consistent with the expression pattern revealed by quantitative real-time PCR analysis. The overexpression of *GmNAC19* enhanced drought tolerance in *Saccharomyces cerevisiae* INVSc1. The seed germination percentage and root growth of transgenic *Arabidopsis thaliana* were improved in comparison with those of the wild type, while the transgenic soybean composite line showed improved chlorophyll content. The altered contents of physiological and biochemical indices (i.e., soluble protein, soluble sugar, proline, and malondialdehyde) related to drought stress and the activities of three antioxidant enzymes (i.e., superoxide dismutase, peroxidase, and catalase) revealed enhanced drought tolerance in both transgenic *Arabidopsis* and soybean. The expressions of three genes (i.e., *P5CS*, *OAT*, and *P5CR*) involved in proline synthesis were decreased in the transgenic soybean hairy roots, while the expression of *ProDH* involved in the breakdown of proline was increased. This study revealed the molecular mechanisms underlying drought tolerance enhanced by *GmNAC19* via regulation of the contents of soluble protein and soluble sugar and the activities of antioxidant enzymes, providing a candidate gene for the molecular breeding of drought-tolerant crop plants.

## 1. Introduction

Soybean is a leguminous crop plant with significant economic and nutritional demand worldwide [1]. Given its wide range of cultivation, soybean growth and development are commonly influenced by a variety of biological and abiotic factors [2]. For example, drought is one of the most important factors influencing soybean yield [1,3,4]. In particular, drought could affect photosynthesis, respiration, and transpiration [5], osmotic regulation [6,7], antioxidant enzyme activity [8], and growth in soybean, thereby altering the growth and development of soybeans and their symbiotic nitrogen fixation, ultimately causing a decrease in soybean yield [1,9]. Hence, the development of drought-tolerant soybean varieties is crucial to meet the growing global demand for soybean [10].

It is well known that plants have evolved a variety of metabolic strategies to cope with abiotic stresses, e.g., drought, while transcription factors (TFs) are commonly involved in the regulation of molecular responses to drought in plants [11]. Due to their regulatory effects on many genes involved in adverse conditions, TFs are generally considered one of the most appropriate genetic targets for the molecular breeding and development of stress-tolerant crop varieties, e.g., inducing drought response in plants [12,13]. Studies have shown that a large number of TFs, e.g., NAC [14], AP2/ERF [15], bZIP [16], bHLH [17], MYB [18,19], and WRKY [20], are frequently involved in the plant response to water stress. In particular, NAC TFs are one of the largest families of plant-specific transcriptional regulators and are widely distributed in plants, including mosses, ferns, gymnosperms, and angiosperms [21,22]. The NAC domain is named after the initial letters of no apical meristem (NAM) of *Petunia*, *Arabidopsis* transcription activation factors (ATAF1 and ATAF2), and cup-shaped cotyledon 2 (CUC2) [23]. Due to their highly conserved N-terminal NAC domains and diverse C-terminal domains, which are involved in the regulation of transcription activities, NAC TFs have been widely studied [23,24]. The N-terminal NAC domain, composed of about 160 amino acids, contains a rare TF folding structure consisting of several helically surrounded β-folds, and structural analysis has identified the functional dimers formed by the N-terminal NAC domain [25]. These functional dimers contain nuclear localization signals and bind to the cis-elements of their target genes. The C-terminal region is highly variable, containing trans-activated regions, and the C-terminal of individual proteins could interact with other proteins to regulate the gene expression, while other domains of NAC TFs contain transmembrane motifs [26]. To date, studies have shown that the NAC TFs of plants play an important role in various biological activities, including plant senescence, cell division, seed development, lateral root formation, and the response to both biotic and abiotic stresses [14,22,27].

Specifically, NAC TF genes in diverse groups of plants, e.g., soybean *GmNAC06* [22], pepper *CaNAC46* [28], potato *SlNAC35* [29], apple *MdNAC1* [30], wheat *TaNAC29* [31], rice *ONAC022* [32], grape *VvNAC17* [33], and chickpea *CarNAC4* [34], play an important role in response to drought stress. For example, the overexpression of *OsNAC066* in rice could improve its drought tolerance, increase the contents of proline and soluble sugar, reduce the accumulation of reactive oxygen species (ROS), and increase the expression of genes involved in stresses [35]. Furthermore, the overexpression of *GmNAC3* [36], *GmNAC4* [37], and *GmNAC8* [38] has enhanced drought tolerance in soybean. These studies have consistently revealed the important regulatory effects of NAC TFs on the plant response to drought stress.

Our previous studies identified the molecular functions of both *GmNAC3* and *GmNAC4* in the drought tolerance of plants [36,37]. The results of transcriptome sequencing analysis of soybean roots showed that *GmNAC19* was significantly up-regulated in response to the treatment of drought stress in soybean. Therefore, we further explored the expression pattern of *GmNAC19* and a group of physiological and biochemical indices related to drought tolerance, as well as the activities of three antioxidant enzymes, to clarify the functions of *GmNAC19* and the molecular mechanism underlying enhanced drought tolerance in soybean. The objectives of this study were to characterize the expression pattern of *GmNAC19*, to detect the variations in the physiological and biochemical factors related to drought tolerance, and to evaluate the activities of three antioxidant enzymes in soybean and *Arabidopsis thaliana*. Our study provided strong experimental evidence to support the function of soybean *GmNAC19* and its molecular response to drought stress in plants, revealing a candidate gene for the potential molecular breeding and development of drought-resistant soybean varieties.

## 2. Results

### 2.1. Expression of Soybean Transcription Factor Gene GmNAC19

#### 2.1.1. Gene Expression of *GmNAC19* Based on Transcriptome Analysis of Drought-Treated Soybean Roots

**Figure 1:**
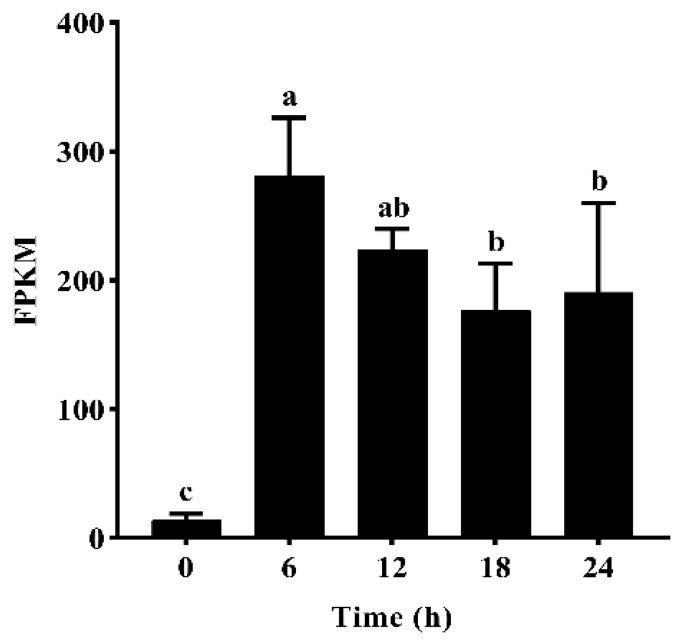
Relative gene expression levels of *GmNAC19* based on transcriptome sequencing analysis of root tips (3 cm in length) of 7-day-old soybean roots treated with 20% PEG6000 for 0, 6, 12, 18, and 24 h. FPKM, fragments per kilobase of transcript per million mapped reads. The statistical significance between two groups was determined by one-way ANOVA, with significant differences in the relative expression level of *GmNAC19* indicated by different lowercase letters (a, b, or c) based on *p* < 0.05 (*n* = 3). The same letters (a, b, or c) indicate no significant difference.

The results of transcriptome sequencing (https://www.ncbi.nlm.nih.gov/sra/; accessed on 30 October 2023; BioProject accession PRJNA1033409) showed that a total of 49 genes in the NAC TF family were significantly up-regulated in soybean roots under drought stress simulated by 20% PEG6000. Specifically, due to its high expression level and the identification of the protein encoded by *GmNAC19* in the NAC domain TF superfamily of proteins, *GmNAC19* was further explored in this study to reveal its molecular functions in response to drought stress in plants. Specifically, the results of the gene expression of *GmNAC19* in soybean roots treated with 20% PEG6000 revealed the highest expression level at 6 h, which was about 20 times higher than that at 0 h (i.e., without the drought treatment). Then, the relative expression level was increased to about 12 and 13.6 times higher than that at 0 h at 18 h and 24 h, respectively (Figure 1).

#### 2.1.2. Gene Expression of *GmNAC19* in Soybean Treated with PEG6000 Based on Quantitative Real-Time PCR

**Figure 2:**
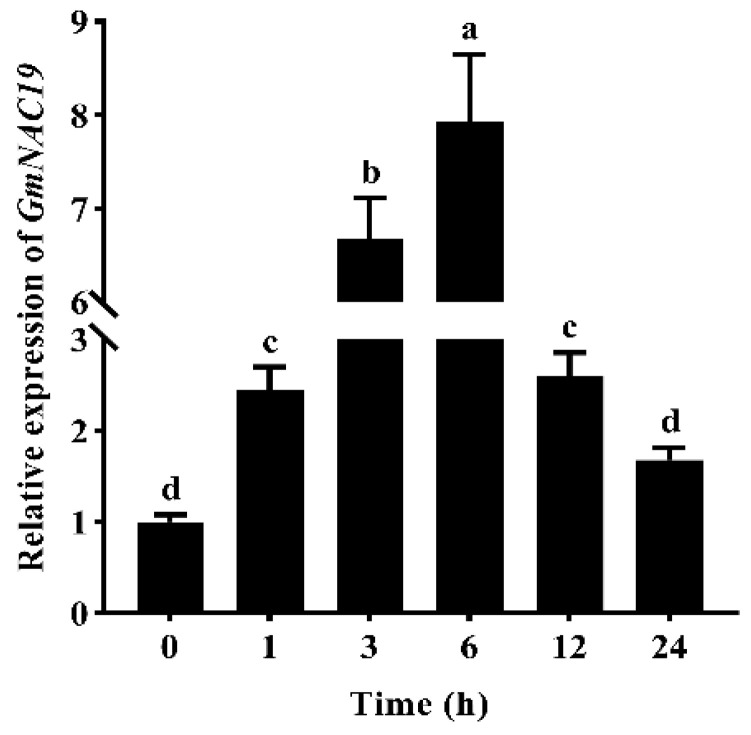
Relative gene expression levels of *GmNAC19* in soybean roots treated with 20% PEG6000 based on qRT-PCR analysis using *GmEF1A* as the reference gene. The statistical significance between two groups was determined by one-way ANOVA, with the significant differences in the relative expression level of *GmNAC19* represented by different lowercase letters (a, b, c, or d) based on *p* < 0.05 (*n* = 3). The same letters (a, b, c, or d) indicate no significant difference.

Based on qRT-PCR, the results of the gene expression of *GmNAC19* in soybean roots treated with 20% PEG6000 showed that the relative gene expression levels of *GmNAC19* were first increased from 0 to 6 h, reaching the highest expression level at 6 h of about 7.9 times higher than that at 0 h, and then, decreased from 6 to 24 h (i.e., about 1.7 times higher than that at 0 h) (Figure 2).

#### 2.1.3. Gene Expression of *GmNAC19* in Different Organs of Soybean at Three Different Developmental Stages

**Figure 3:**
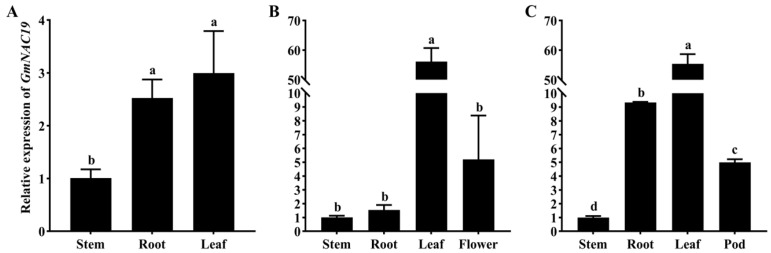
Relative gene expression levels of *GmNAC19* in different organs (i.e., stems, roots, leaves, flowers, and pods) of soybean plants at three developmental stages, i.e., seedling (**A**), flowering (**B**), and podding (**C**) based on qRT-PCR analysis using *GmEF1A* as the reference gene. The statistical significance between two groups was determined by one-way ANOVA, with the significant differences in the relative expression level of *GmNAC19* represented by different lowercase letters (a, b, c, or d) based on *p* < 0.05. The same letters (a, b, c, or d) indicate no significant difference.

The relative expression levels of *GmNAC19* were further detected in different organs (i.e., stems, roots, leaves, flowers, and pods) of soybean plants at three developmental stages, i.e., seedling, flowering, and podding (Figure 3). The results showed that among the different organs, the leaves were consistently revealed to have the highest relative expression levels of *GmNAC19* during the three developmental stages, whereas the lowest relative expression levels were consistently detected in the stems. Specifically, the relative expression levels in leaves were 2.9, 56, and 55 times higher than those in the stems during the seeding, flowering, and podding stages, respectively. During the seedling stage (Figure 3A), the relative expression levels of *GmNAC19* were significantly different between the stems and roots and between the stems and leaves, whereas no significant difference was detected between the roots and leaves. During the flowering stage (Figure 3B), no significant differences were detected among the stems, roots, and flowers, whereas during the podding stage (Figure 3C), significant differences were detected among all four organs examined.

### 2.2. Molecular Mechanism Underlying the Enhancement in Drought Tolerance by Soybean GmNAC19

#### 2.2.1. Enhanced Drought Tolerance in *Saccharomyces cerevisiae* Conferred by Soybean *GmNAC19*

**Figure 4:**
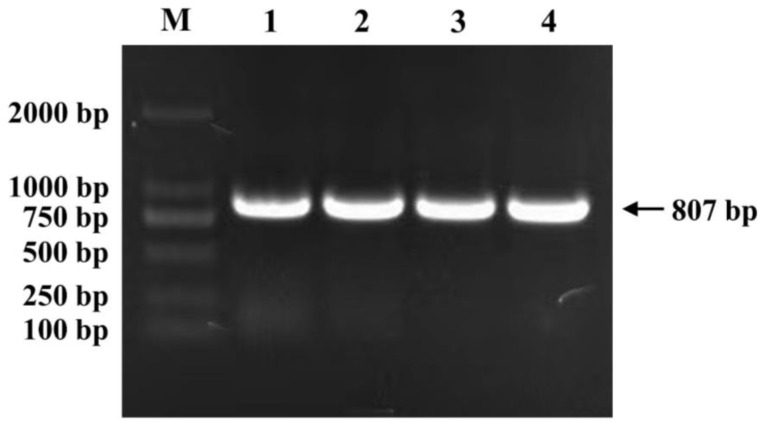
Verification of pYES2-*GmNAC19* yeast expression vector using bacterial solution of *Escherichia coli* (Lanes 1 and 2) and plasmid (Lanes 3 and 4) PCR analyses. Lane M, DL2000 Marker.

To construct the pYES2-*GmNAC19* yeast expression vector, the amplified target PCR product was connected with a pYES2 yeast expression vector and transformed into competent cells of *Escherichia coli* DH5α. A single colony was selected for further culture and plasmid extraction. The successful construction of the pYES2-*GmNAC19* yeast expression vector was verified using a bacterial solution and plasmid PCR analyses based on the target PCR-amplified products and sequences obtained (Figure 4).

**Figure 5:**
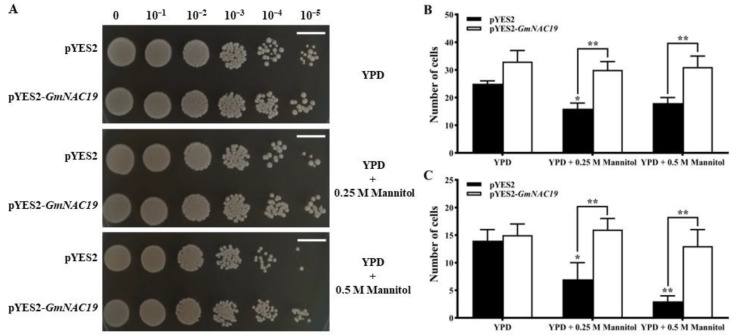
Drought tolerance in control (pYES2) and transgenic (pYES2-*GmNAC19*) *Saccharomyces cerevisiae* with soybean *GmNAC19* under the treatment of mannitol with two concentrations (0.25 M and 0.5 M) at 48 h. (**A**) Morphological observations of yeast growth based on various yeast inoculation dilutions. Scale bar = 1 cm. (**B**) Number of yeast cells in the control (pYES2) and transgenic (pYES2-*GmNAC19*) groups based on yeast inoculation dilution of 10⁻^4^ CFU/mL. (**C**) Number of yeast cells in the control (pYES2) and transgenic (pYES2-*GmNAC19*) groups based on yeast inoculation dilution of 10⁻^5^ CFU/mL. One-way ANOVA followed by post hoc Tukey’s test was performed to evaluate the significant differences in the number of yeast cells between the control without treatment of mannitol and controls with treatment of mannitol, and between transgenic group without treatment of mannitol and transgenic groups with treatment of mannitol; two-way ANOVA tests were performed to detect significant differences in the number of yeast cells between the control and transgenic groups cultured on the same type of medium (indicated by asterisk above the bracket) based on *p* < 0.05 (*) or *p* < 0.01 (**) (*n* = 3).

The drought tolerance in transgenic yeast with *GmNAC19* was further evaluated. The results showed that on YPD medium, the yeast in both the control and the transgenic groups could grow normally, showing no significant variations in their development (Figure 5A). With the increase in mannitol content in the YPD medium, the growth of yeast in both the control and the transgenic groups was decreased, whereas the transgenic group showed improved growth performance, as observed in the increased cell number (Figure 5B,C). These results indicated that transgenic yeast with *GmNAC19* gained enhanced drought tolerance.

#### 2.2.2. Enhanced Drought Tolerance in Transgenic *Arabidopsis thaliana* with *GmNAC19*

- Drought tolerance of transgenic *Arabidopsis thaliana* with *GmNAC19* under drought stress simulated by PEG6000

(1) Seed germination of transgenic *Arabidopsis thaliana* with *GmNAC19*

**Figure 6:**
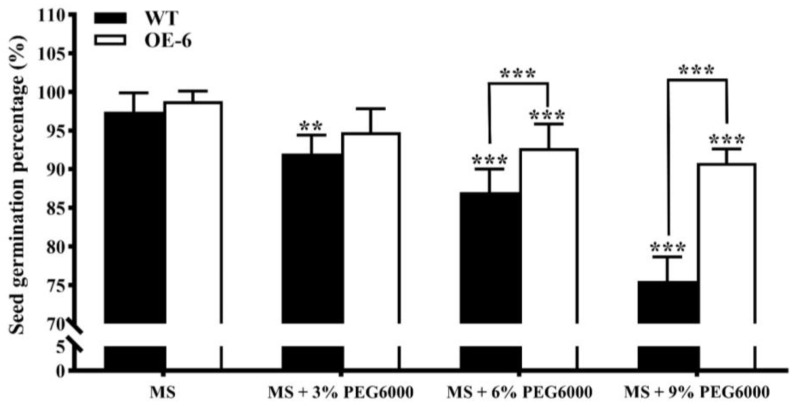
Seed germination of wild-type (WT) and transgenic *Arabidopsis thaliana* with *GmNAC19* (OE-6) treated with PEG6000 at 0%, 3%, 6%, and 9% for 15 d. Each treatment group contains 3 replicates (64 seeds in each replicate). One-way ANOVA followed by post hoc Tukey’s test was performed to evaluate the significant differences in seed germination percentage between WT without treatment of PEG6000 and WT with treatment of PEG6000, and between transgenic group without treatment of PEG6000 and transgenic groups with treatment of PEG6000; two-way ANOVA tests were performed to detect the significant differences in seed germination percentage between WT and transgenic groups in the same treatment group (indicated by asterisk above the bracket) based on *p* < 0.01 (**) and *p* < 0.001 (***) (*n* = 3).

The seed germination percentages of both the wild-type (WT) and one line (OE-6) of T3 generation transgenic *A. thaliana* with *GmNAC19* were evaluated based on seeds sown on MS medium with PEG6000 at 0%, 3%, 6%, and 9% in 15 d (Figure 6). The results showed that the average germination percentages of WT *A. thaliana* were revealed to have a largely decreasing pattern, reaching 97.4%, 92.02%, 86.98%, and 75.52% as the concentrations of PEG6000 were increased, i.e., 0%, 3%, 6%, and 9%, respectively. Under the treatments of PEG6000 at 6% and 9%, the seed germination percentages of transgenic *A. thaliana* with *GmNAC19* (95.31% and 93.23%) were significantly higher than those of WT (86.98% and 75.52%, respectively).

(2) Growth of roots in transgenic *Arabidopsis thaliana* with *GmNAC19*

**Figure 7:**
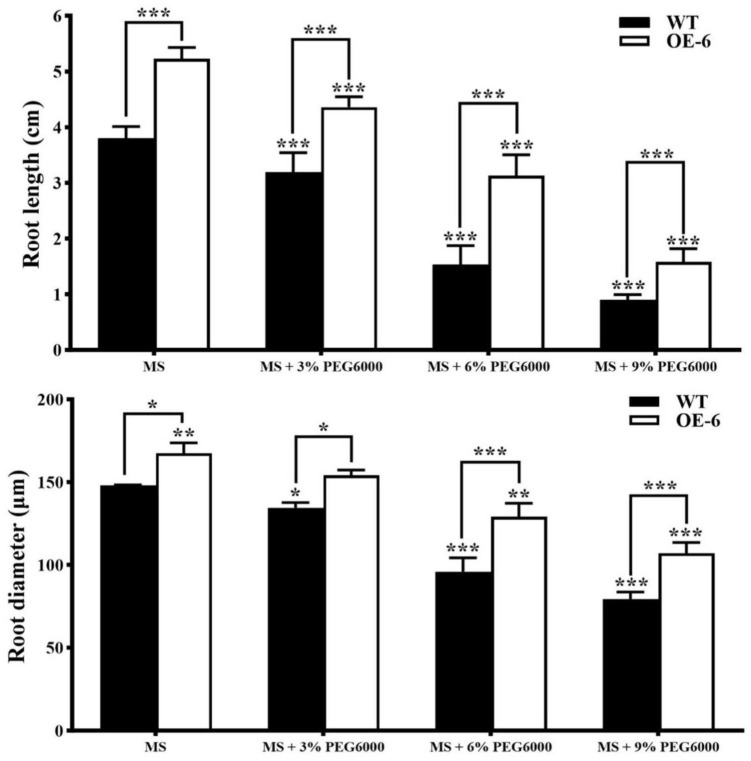
Growth of roots of wild-type (WT) and transgenic *Arabidopsis thaliana* of *GmNAC19* (OE-6) treated with PEG6000 at 0%, 3%, 6%, and 9%, for 15 d. One-way ANOVA followed by post hoc Tukey’s test was performed to evaluate the significant differences in root length and root diameter between WT without treatment of PEG6000 and WT with treatment of PEG6000, and between transgenic group without treatment of PEG6000 and transgenic groups with treatment of PEG6000; two-way ANOVA tests were performed to detect the significant differences in root length and root diameter between WT and transgenic groups in the same treatment group (indicated by asterisk above the bracket) based on *p* < 0.05 (*), *p* < 0.01 (**), and *p* < 0.001 (***) (*n* = 3 with each replicate of 5 plants).

Seeds of both WT and one line (OE-6) of T3 generation transgenic *A. thaliana* of *GmNAC19* were sown on MS medium with PEG6000 at 0%, 3%, 6%, and 9%, with the root length measured in a total of 15 plants (three replicates each of five plants) of each group in 15 d (Figure 7). The results showed that the average root lengths of WT *A. thaliana* were 3.8 cm, 3.19 cm, 1.53 cm, and 0.9 cm on MS medium containing PEG6000 at 0%, 3%, 6%, and 9%, respectively, which were significantly shorter than those of transgenic *A. thaliana* with *GmNAC19*, i.e., 5.23 cm, 4.36 cm, 3.13 cm, and 1.58 cm, respectively. These results showed that the root elongation in *A. thaliana* was inhibited by an increased concentration of PEG6000, whereas the root growth in transgenic *A. thaliana* with *GmNAC19* grown in MS medium with different concentrations of PEG6000 was significantly improved in comparison with that of WT.

To summarize, the results of the seed germination and root growth of transgenic *A. thaliana* with *GmNAC19* treated with PEG6000 at various concentrations showed that as the level of drought stress was increased, the germination percentage of transgenic *A. thaliana* remained above 93%, whereas the germination percentage of WT was significantly decreased to 86.98% (at 6% PEG6000) and 75.52% (at 9% PEG6000). The largest increase of 1.6 cm was detected from the average root lengths of WT to transgenic *A. thaliana* with *GmNAC19* grown under the treatment of 6% PEG6000. Therefore, the treatment of 6% PEG6000 with a high seed germination percentage maintained was selected for the subsequent evaluation of physiological and biochemical indices related to drought tolerance.

The results of phenotypic variations revealed no significant difference in the growth of WT and transgenic *A. thaliana* under the normal growth conditions, whereas the wilting degree of the WT was significantly higher than that of the transgenic plants under drought stress (i.e., treatment of 6% PEG6000), and the recovery ability of the transgenic plants was stronger than that of the WT after re-watering (Figure S1). Furthermore, the results of the 100-seed weight showed that the 100-seed weights of the transgenic plants were significantly higher than those of the WT (Table 1).

(3) Contents of physiological and biochemical indices related to drought stress in transgenic *Arabidopsis thaliana* with *GmNAC19* under drought stress simulated by PEG6000

**Figure 8:**
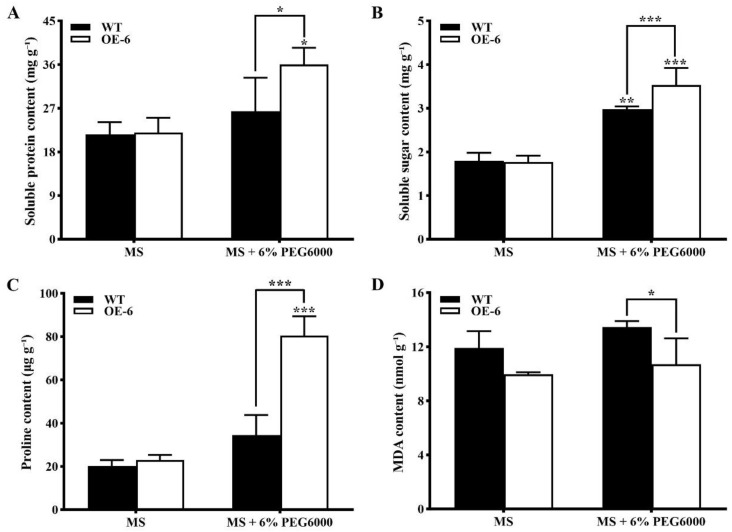
Contents of physiological and biochemical indices, i.e., soluble protein (**A**), soluble sugar (**B**), proline (**C**), and malondialdehyde (MDA) (**D**), in wild-type (WT) and transgenic *Arabidopsis thaliana* with *GmNAC19* (OE-6) under drought stress simulated by 6% PEG6000 for 15 d. One-way ANOVA followed by post hoc Tukey’s test was performed to evaluate the significant differences in the content of each index between WT without treatment of PEG6000 and WT with treatment of PEG6000, and between transgenic group without treatment of PEG6000 and transgenic group with treatment of PEG6000; two-way ANOVA tests were performed to detect the significant differences in the content of each index between WT and transgenic groups in the same treatment group based on *p* < 0.05 (*), *p* < 0.01 (**), and *p* < 0.001 (***) (*n* = 3).

On MS medium without PEG6000, no significant differences in the contents of soluble protein, soluble sugar, proline, and malondialdehyde (MDA) were detected between the WT and transgenic *A. thaliana* with *GmNAC19* (Figure 8). On MS medium containing 6% PEG6000, the contents of soluble protein, proline, and soluble sugar were significantly higher in the transgenic *A. thaliana* with *GmNAC19* than those of the WT (Figure 8A–C), while a significant decrease was detected in the content of MDA between the WT and transgenic *Arabidopsis thaliana* with *GmNAC19* (Figure 8D).

(4) Activities of antioxidant enzymes in transgenic *Arabidopsis thaliana* with *GmNAC19* under drought stress simulated by PEG6000

**Figure 9:**
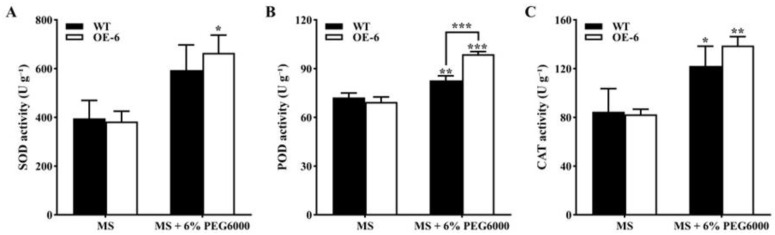
Antioxidant enzyme activities of superoxide dismutase (SOD) (**A**), peroxidase (POD) (**B**), and catalase (CAT) (**C**), in wild-type (WT) and transgenic *Arabidopsis thaliana* of *GmNAC19* (OE-6) under drought stress simulated by 6% PEG6000 for 15 d. One-way ANOVA followed by post hoc Tukey’s test was performed to evaluate the significant differences in the enzymatic activities between WT without treatment of PEG6000 and WT with treatment of PEG6000, and between transgenic group without treatment of PEG6000 and transgenic group with treatment of PEG6000; two-way ANOVA tests were performed to detect the significant differences in the enzymatic activities between WT and transgenic groups in the same treatment group (indicated by asterisk above the bracket) based on *p* < 0.05 (*), *p* < 0.01 (**), and *p* < 0.001 (***) (*n* = 3). Note: A significant increase is detected in the enzymatic activities of SOD, POD, and CAT in transgenic *A. thaliana* on MS medium containing PEG6000 compared with WT on MS without PEG6000 based on Student’s *t* test (*p* < 0.05).

There was no significant difference in the activities of the three antioxidant enzymes on MS medium without PEG6000 between the WT and transgenic *A. thaliana* with *GmNAC19* (Figure 9). On MS medium with PEG6000, superoxide dismutase (SOD) showed higher activity in the transgenic group than that on MS medium without PEG6000 (Figure 9A). On MS medium containing 6% PEG6000, the enzymatic activity of peroxidase (POD) was higher in the transgenic *A. thaliana* with *GmNAC19* than that in the WT (Figure 9B), while higher activities of both POD and catalase (CAT) were observed in the WT and transgenic groups than those on MS medium without PEG6000 (Figure 9B,C). It was noted that a significant increase was detected in the enzymatic activities of SOD, POD, and CAT in transgenic *A. thaliana* on MS medium containing PEG6000 compared with the WT on MS without PEG6000.

- Drought tolerance of transgenic *Arabidopsis thaliana* with *GmNAC19* under drought stress induced by water loss

(1) Contents of physiological and biochemical indices related to drought stress of transgenic *Arabidopsis thaliana* with *GmNAC19* under drought stress induced by water loss

**Figure 10:**
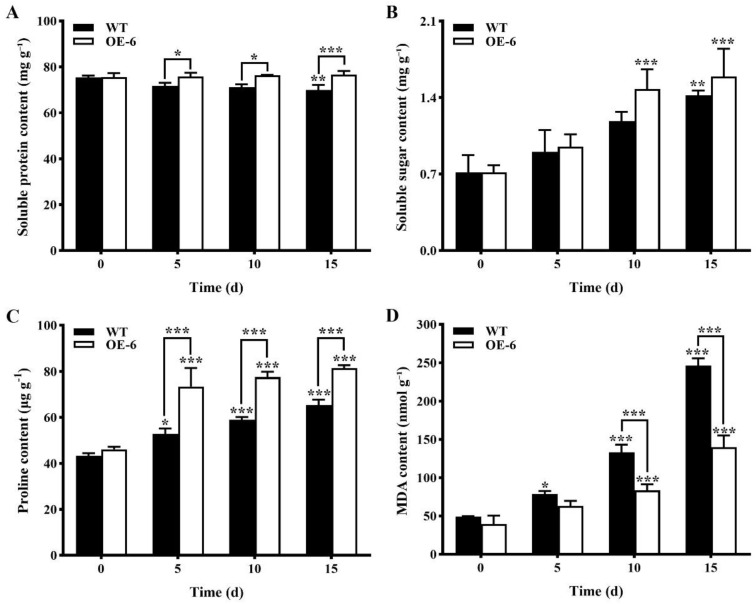
Contents of physiological and biochemical indices, i.e., soluble protein (**A**), soluble sugar (**B**), proline (**C**), and malondialdehyde (MDA) (**D**), related to drought stress in wild-type (WT) and transgenic *Arabidopsis thaliana* with *GmNAC19* (OE-6) under drought stress induced by water loss. One-way ANOVA followed by post hoc Tukey’s test was performed to evaluate the significant differences in the content of each index between WT at 0 d and WT at 5, 10, and 15 d, and between transgenic group at 0 d and transgenic groups at 5, 10, and 15 d; two-way ANOVA tests were performed to detect the significant differences in the content of each index between WT and transgenic groups at the same sampling time point (indicated by asterisk above the bracket) based on *p* < 0.05 (*), *p* < 0.01 (**), and *p* < 0.001 (***) (*n* = 3). Note: In 15 d, a significant increase in the content of soluble sugar (**B**) is detected in transgenic *A. thaliana* under drought stress compared with WT without drought stress (0 d) based on Student’s *t* test (*p* < 0.05).

Significant variations in the content of soluble protein were observed between WT and transgenic *A. thaliana* under drought stress; in 15 d, the content of soluble protein was decreased in the WT, with an average of 72.1 mg g^−1^, which was significantly lower than that of the transgenic group of *A. thaliana*, with an average of 75.9 mg g^−1^ (Figure 10A). In 15 d, the contents of soluble sugar in the WT and transgenic groups were significantly increased by 0.70 mg g^−1^ and 0.87 mg g^−1^, respectively, in comparison to the WT without drought stress (Figure 10B), and it was noted that a significant increase in the content of soluble sugar was detected in transgenic *A. thaliana* under drought stress compared with the WT without drought stress. Under drought stress, a significant increase was detected in the proline content of transgenic *A. thaliana* compared with the WT (Figure 10C); these results were consistent with the above results based on the treatment of drought stress simulated by PEG6000. The contents of MDA were found to exhibit an increasing pattern in both the WT and transgenic groups, with the contents of MDA in the transgenic group significantly lower than those of the WT at 10 and 15 d, respectively, under drought stress (Figure 10D).

(2) Activities of antioxidant enzymes of transgenic *Arabidopsis thaliana* with *GmNAC19* under drought stress induced by water loss

**Figure 11:**
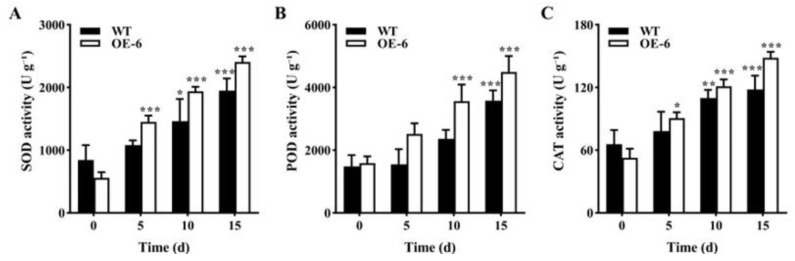
Antioxidant enzyme activities of superoxide dismutase (SOD) (**A**), peroxidase (POD) (**B**), and catalase (CAT) (**C**) in wild-type (WT) and transgenic *Arabidopsis thaliana* with *GmNAC19* (OE-6) under drought stress induced by water loss. One-way ANOVA followed by post hoc Tukey’s test was performed to evaluate the significant differences in the enzymatic activity between WT at 0 d and WT at 5, 10, and 15 d, and between transgenic group at 0 d and transgenic groups at 5, 10, and 15 d; two-way ANOVA tests were performed to detect the significant differences in the enzymatic activity between WT and transgenic groups at the same sampling time point (indicated by asterisk above the bracket) based on *p* < 0.05 (*), *p* < 0.01 (**), and *p* < 0.001 (***) (*n* = 3). Note: In 15 d, the enzymatic activities of SOD, POD, and CAT are significantly increased in the transgenic groups compared with the WT without drought stress (0 d) based on Student’s *t* test (*p* < 0.05).

With the extension of drought stress treatment time, the enzymatic activities of SOD, POD, and CAT in both the WT and transgenic groups of *A. thaliana* were generally increased, reaching the highest levels at 15 d (Figure 11). At 15 d, the levels of enzymatic activities of SOD, POD, and CAT in the transgenic group were increased by 1847.7, 2902.3, and 95.8 U g^−1^, respectively, which were higher than those in the WT, i.e., 1104.4, 2095.3, and 52.4 U g^−1^, respectively. It was noted that at 15 d, the enzymatic activities of SOD, POD, and CAT were significantly increased in the transgenic groups compared with the WT without drought stress.

#### 2.2.3. Enhanced Drought Tolerance in Transgenic Soybean Composite Lines with *GmNAC19*

- Content of chlorophyll in transgenic soybean composite lines with *GmNAC19* under drought stress simulated by 20% PEG6000

**Figure 12:**
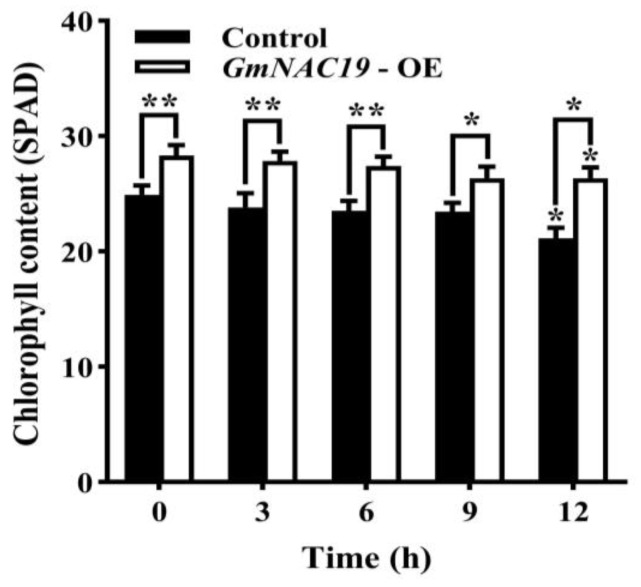
Chlorophyll content in the control and transgenic soybean composite lines with *GmNAC19* (OE) under drought stress simulated by 20% PEG6000. The control group contains plants transformed with *Agrobacterium rhizogenes* K599 without *GmNAC19*. One-way ANOVA followed by post hoc Tukey’s test was performed to evaluate the significant differences in the chlorophyll content between control at 0 h and controls at 3, 6, 9, and 12 h, and between transgenic group at 0 h and transgenic groups at 3, 6, 9, and 12 h; two-way ANOVA tests were performed to detect the significant differences in the chlorophyll content between control and transgenic groups at the same sampling time point (indicated by asterisk above the bracket) based on *p* < 0.05 (*) and *p* < 0.01 (**) (*n* = 3).

As the treatment time of drought stress was increased from 0 to 9 h, the chlorophyll contents of both the control group and the transgenic soybean composite lines with *GmNAC19* showed no significant variation (Figure 12). At 12 h, the chlorophyll contents of both control group and transgenic soybean composite lines with *GmNAC19* were significantly lower than those without drought stress (0 d), respectively. The transgenic group was revealed to have significantly higher chlorophyll content than the control group at all sampling time points.

- Contents of physiological and biochemical indicators related to drought stress in transgenic soybean composite lines with *GmNAC19* under drought stress simulated by 20% PEG6000

**Figure 13:**
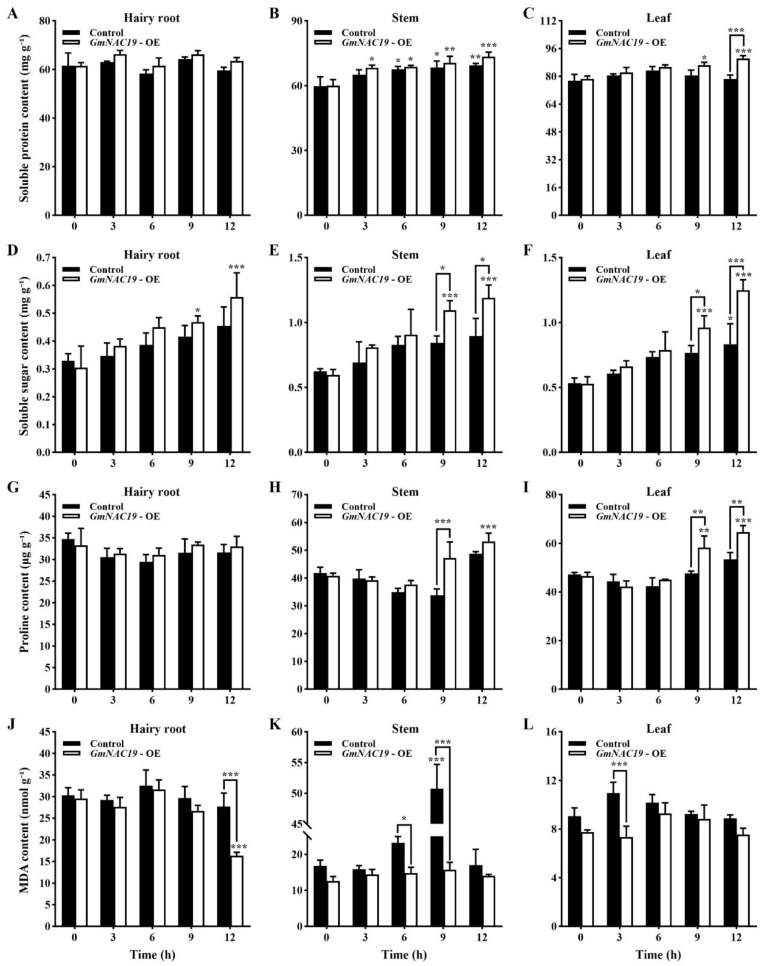
Contents of physiological and biochemical indicators, i.e., soluble protein (**A**–**C**), soluble sugar (**D**–**F**), proline (**G**–**I**), and malondialdehyde (MDA) (**J**–**L**) related to drought stress in hairy roots, stems, and leaves of the control and the transgenic soybean composite lines with *GmNAC19* (OE) under drought stress simulated by 20% PEG6000. The control groups contain plants transformed with *Agrobacterium rhizogenes* K599 without *GmNAC19*. One-way ANOVA followed by post hoc Tukey’s test was performed to evaluate the significant differences in the content of each index between control at 0 h and controls at 3, 6, 9, and 12 h, and between transgenic group at 0 h and transgenic groups at 3, 6, 9, and 12 h; two-way ANOVA tests were performed to detect the significant differences in the content of each index between control and transgenic groups at the same sampling time point (indicated by asterisk above the bracket) based on *p* < 0.05 (*), *p* < 0.01 (**), and *p* < 0.001 (***) (*n* = 3). Note: At 12 h, a significant difference is detected between the transgenic plant and the control without drought stress in the content of soluble protein in the leaves, in the content of soluble sugar in all three organs, in the proline content in the stems and leaves, and in the content of MDA in both the roots and leaves based on Student’s *t* test (*p* < 0.05).

No significant differences were detected in the contents of soluble protein in hairy roots in the control group and the transgenic soybean composite lines with *GmNAC19*, while as the treatment time was increased, significant variations in the contents of soluble protein were observed in the stems and leaves of the control and the transgenic soybean composite lines with *GmNAC19* (Figure 13A–C). At 12 h, the content of soluble protein in the leaves of the transgenic group was significantly higher than that of the control.

No significant variations were revealed in the contents of soluble sugar in the hairy roots and stems in the control group, while a significant difference was observed in stems and leaves of control group at 9 and 12 h, respectively (Figure 13D–F). As the treatment time of drought stress was increased to 9 and 12 h, the soluble sugar content in the hairy roots, stems, and leaves of the transgenic groups were significantly higher than that at 0 h. At 9 and 12 h, the contents of soluble sugar in the stems and leaves of the transgenic group were significantly higher than those of the control group.

No significant differences were detected in the contents of proline in the hairy roots, stems, and leaves of the control group, while the transgenic group showed significant variations in the stems (at 12 h) and leaves (at 9 and 12 h) (Figure 13G–I). Significant variations were revealed in the stems (at 9 h) and leaves (at 9 and 12 h) between the control and the transgenic groups.

The contents of MDA were generally not significantly different in the hairy roots, stems, and leaves of the control and the transgenic groups, except for the hairy roots (at 12 h) in the transgenic group and the stems (at 9 h) in the control group (Figure 13J–L). Significant variations were revealed in the hairy roots (at 12 h), stems (at 6 and 9 h), and leaves (at 3 h) between the control and the transgenic groups.

- Antioxidant enzyme activities of transgenic soybean composite lines with *GmNAC19* under drought stress simulated by 20% PEG6000

**Figure 14:**
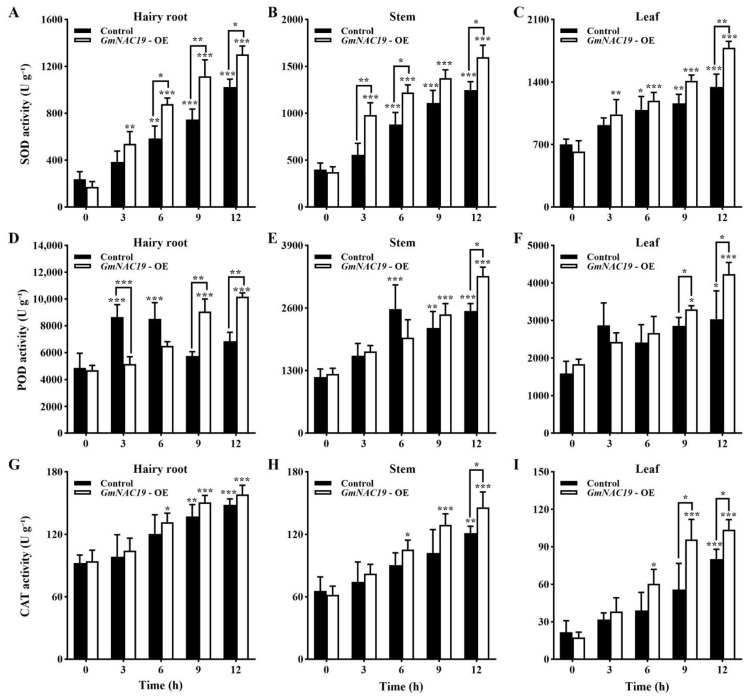
Antioxidant enzyme activities of superoxide dismutase (SOD) (**A**–**C**), peroxidase (POD) (**D**–**F**), and catalase (CAT) (**G**–**I**) in the hairy roots, stems, and leaves of transgenic soybean composite lines with *GmNAC19* (OE) under drought stress simulated by 20% PEG6000. The control groups contain the plants transformed with *Agrobacterium rhizogenes* K599 without *GmNAC19*. One-way ANOVA followed by post hoc Tukey’s test was performed to evaluate the significant differences in the enzymatic activity between control at 0 h and controls at 3, 6, 9, and 12 h, and between transgenic group at 0 h and transgenic groups at 3, 6, 9, and 12 h; two-way ANOVA tests were performed to detect the significant differences in the enzymatic activity between control and transgenic groups at the same sampling time point (indicated by asterisk above the bracket) based on *p* < 0.05 (*), *p* < 0.01 (**), and *p* < 0.001 (***) (*n* = 3). Note: At 12 h, the enzymatic activities of SOD, POD, and CAT in all three organs of transgenic plants are significantly higher than those of the controls without drought stress (0 d) based on Student’s *t* test (*p* < 0.05).

As the treatment time of drought stress was increased, the SOD activities in the hairy roots, stems, and leaves of both the control and the transgenic groups showed an increasing pattern, showing significantly higher SOD activities in the hairy roots (at 6, 9, and 12 h), stems (at 3, 6, and 12 h), and leaves (at 12 h) of the transgenic group than those in the control group (Figure 14A–C). At 12 h, the SOD activities in the hairy roots, stems, and leaves were increased to 4.3, 3.1, and 1.9 times higher than those without the treatment of drought stress in the control group, and 7.7, 4.3, and 2.9 times higher than those without the treatment of drought stress in the transgenic group, respectively.

The highest levels of enzymatic activities of POD were detected in the hairy roots, followed by the leaves and stems (Figure 14D–F). In the hairy roots, the enzymatic activities of POD were first increased (reaching the highest levels at 3 and 6 h, which was 1.78 times higher than that at 0 h), and then, decreased (at 9 and 12 h) in the control group, whereas the transgenic group showed an increasing pattern; significant differences were revealed in the hairy roots between the control and transgenic groups at 3, 9, and 12 h. In both the stems and leaves, with an increase in the PEG6000 treatment time, the POD activities were significantly increased in the stems (at 6, 9, and 12 h in the control and at 9 and 12 h in the transgenic group) and leaves (at 12 h in the control and at 9 and 12 h in the transgenic group). At 12 h, the POD activities in the hairy roots, stems, and leaves of the transgenic group were 1.5, 1.3, and 1.4 times higher than those in the control group.

With an increase in the PEG6000 treatment time, the enzymatic activities of CAT in the hairy roots, stems, and leaves were generally increased in both the control and transgenic groups (Figure 14G–I). A significant difference in the enzymatic activity of CAT was observed in the leaves between the control and the transgenic groups at 9 and 12 h. At 12 h, the CAT activities were increased by 56.2, 55.4, and 58.6 U g^−1^ in the hairy roots, stems, and leaves, respectively, in the control group, and by 64.2, 83.9, and 86.3 U g^−1^, respectively, in the transgenic group. It was noted that at 12 d, the enzymatic activities of SOD, POD, and CAT in all three organs of the transgenic plants were significantly higher than those of the controls without drought stress (0 d).

- Gene expression of *GmNAC19* in soybean hairy roots under drought stress simulated by 20% PEG6000

**Figure 15:**
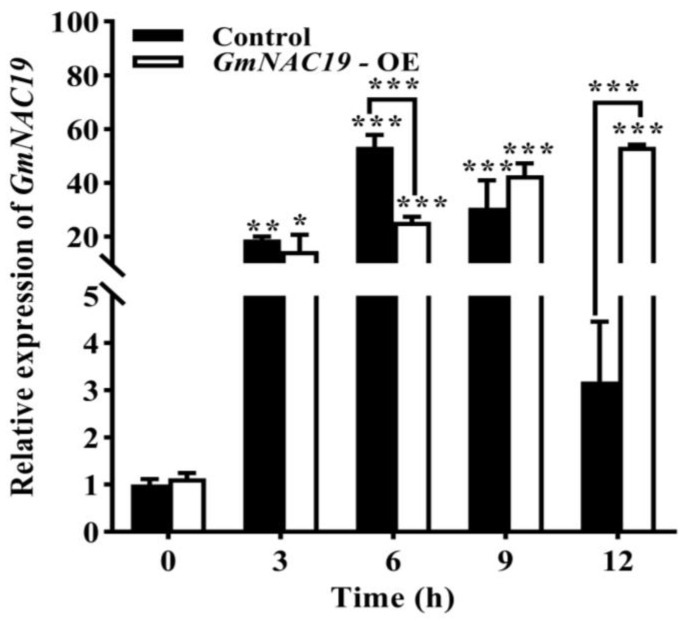
Relative expression of *GmNAC19* in transgenic soybean hairy roots with *GmNAC19* (OE) under drought stress simulated by 20% PEG6000. The control group contains the soybean hairy roots transformed with *Agrobacterium rhizogenes* K599 without *GmNAC19*. One-way ANOVA followed by post hoc Tukey’s test was performed to evaluate the significant differences in the relative expression of *GmNAC19* between control at 0 h and controls at 3, 6, 9, and 12 h, and between transgenic group at 0 h and transgenic groups at 3, 6, 9, and 12 h; two-way ANOVA tests were performed to detect the significant differences in the relative expression of *GmNAC19* between control and transgenic groups at the same sampling time point (indicated by asterisk above the bracket) based on *p* < 0.05 (*), *p* < 0.01 (**), and *p* < 0.001 (***) (*n* = 3).

Under the treatment of drought stress, the expression levels of *GmNAC19* in the control group were first increased to the highest level at 6 h, and then, decreased (at 12 h) (Figure 15), which was consistent with the expression pattern of *GmNAC19* revealed by the transcriptome sequencing and qRT-PCR analyses (Figure 1 and Figure 2), showing that the gene expression levels of *GmNAC19* in soybean roots treated with 20% PEG6000 were first increased from 0 to 6 h, reaching the highest expression level at 6 h, and then, decreased from 6 to 24 h (Figure 2). In the transgenic group, the gene expression of *GmNAC19* was gradually increased and reached the highest level at 12 h, which was 53 and 47 times higher than that of the control and the transgenic groups without the treatment of drought stress (at 0 h), respectively.

- Expression of four key genes involved in proline metabolic pathway in transgenic soybean hairy roots with *GmNAC19* under drought stress simulated by 20% PEG6000

**Figure 16:**
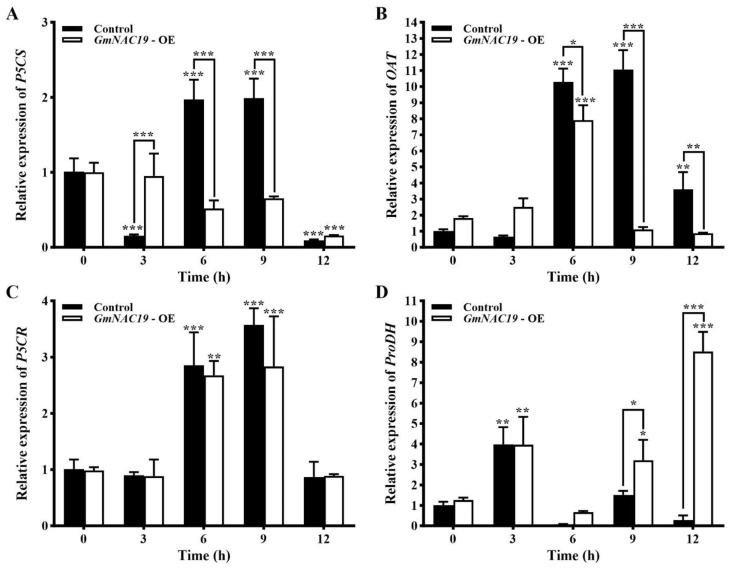
Relative expression levels of genes, i.e., *P5CS* (**A**), *OAT* (**B**), *P5CR* (**C**), and *ProDH* (**D**), involved in proline metabolic pathway in soybean hairy roots with *GmNAC19* (OE) under drought stress simulated by 20% PEG6000. The control groups contain soybean hairy roots transformed with *Agrobacterium rhizogenes* K599 without *GmNAC19*. One-way ANOVA followed by post hoc Tukey’s test was performed to evaluate the significant differences in the relative expression of each gene between control at 0 h and controls at 3, 6, 9, and 12 h, and between transgenic group at 0 h and transgenic groups at 3, 6, 9, and 12 h; two-way ANOVA tests were performed to detect the significant differences in the relative expression of each gene between control and transgenic groups at the same sampling time point (indicated by asterisk above the bracket) based on *p* < 0.05 (*), *p* < 0.01 (**), and *p* < 0.001 (***) (*n* = 3).

Varied expression patterns were observed in the four key genes, i.e., *P5CS*, *OAT*, *P5CR*, and *ProDH*, involved in the proline metabolic pathway (Figure 16). The expression levels of *P5CS* in the control group were first decreased (at 3 h), and then, increased to the highest levels (at 6 and 9 h), and finally, decreased (at 12 h), whereas significant variation was only detected at 12 h in the transgenic group (Figure 16A); the expression levels of *P5CS* showed significant differences between the control and transgenic groups at 3, 6, and 9 h. The same expression pattern of *OAT* was revealed in both the control and transgenic groups, i.e., first increased (at 6 and 9 h in the control group and at 6 h in the transgenic group), and then, decreased (at 12 h in the control group and at 9 and 12 h in the transgenic group) (Figure 16B); the expression levels of *OAT* showed significant differences between the control and transgenic groups at 6, 9, and 12 h. The expression patterns of *P5CR* were largely the same as those of *OAT*, showing that the expression levels of *P5CR* were first increased (at 6 and 9 h), and then, decreased (at 12 h) in both the control and transgenic groups (Figure 16C). The expression levels of *ProDH* were first increased (at 3 h, reaching the highest level, which was 3.95 times higher than that of the control group at 0 h), and then, decreased (at 6, 9, and 12 h) in the control group, whereas in the transgenic group, the expression levels of *ProDH* were first increased (at 3 h), and then, decreased at 6 h, and finally, increased (at 12 h, reaching the highest level, which was 8.46 times higher than that of the control group at 0 h) (Figure 16D); the expression levels of *ProDH* showed a significant difference between the control and transgenic groups at 9 and 12 h.

- Correlation analysis between gene expression levels of *GmNAC19* and four key genes involved in proline metabolic pathway

Pearson correlation analyses between the gene expression level of *GmNAC19* (Figure 15) and the expression levels of the four key genes (Figure 16) involved in proline metabolism in soybean hairy roots were performed in both the control and the transgenic groups (Table 2). The results showed that in the control group, the gene expression level of *GmNAC19* was significantly positively correlated with that of the three genes (i.e., *P5CS*, *OAT*, and *P5CR*) involved in the synthesis of proline and was not significantly correlated with that of the gene *ProDH* involved in the breakdown of proline. In the transgenic group, the gene expression level of *GmNAC19* was significantly positively and negatively correlated with that of *ProDH* and *P5CS*, respectively.

## 3. Discussion

### 3.1. Gene Expression Patterns of Soybean Transcription Factor GmNAC19

In response to drought stress, drought tolerance genes in plants are expressed and have been revealed to have varied expression levels and activities involved in many metabolic pathways to enhance drought tolerance in plants [13,39,40,41]. TFs are important and indispensable regulatory proteins involved in numerous biological activities in plants, including drought tolerance. In particular, NAC TFs play a key role in the molecular mechanism underlying drought tolerance in plants in response to drought stress by regulating the expression of related genes at the transcriptional level [28,29,30,31,32,33,34,35,36,37,38].

To date, many *NAC* genes have been revealed to exhibit enhanced expression under drought stress in many model plants, including rice, *Arabidopsis*, and soybean [36,37,38,42]. In our study, the results of transcriptome sequencing analysis of the soybean variety ‘Jiyu47’ under drought stress revealed significant up-regulation in the expression of the TF gene *GmNAC19*, reaching the highest level (about 20 times higher than that without the treatment of drought stress) at 6 h under the treatment of drought stress, and then, increased to 12 times higher than that without the treatment of drought stress at 18 h, and finally, increased to 13.6 times higher than that without the treatment of drought stress at 24 h. These results were consistent with those derived from the transgenic soybean hairy roots under drought stress simulated by PEG6000. It is worth noting that the different NAC genes located in the nucleus showed varied molecular responses to drought stress (Table 3). For example, both *GmNAC3* and *GmNAC4* responded to drought stress mainly by increasing the enzymatic activities of SOD and CAT, as well as increasing the proline content, while *GmNAC8* responded to drought stress by increasing the enzymatic activity of POD and the proline content [36,37,38]. Our results showed that *GmNAC19* responded to drought stress by not only significantly increasing the enzymatic activities of POD, SOD, and CAT and the chlorophyll content of soybeans, but also regulating chlorophyll content to maintain photosynthesis, respiration, and material accumulation, further influencing plant growth and development, and ultimately enhancing drought resistance in soybean plants.

Various expression patterns have been reported in many genes in response to drought stress simulated by PEG6000. In our study, the gene expression of *GmNAC19* detected in hairy roots prior to the treatment of 20% PEG6000 was first increased, and then, decreased, reaching the highest expression level at 6 h, which was 8 times higher than that at 0 h. These results were consistent with those of the expression patterns of many TFs under drought stress. For example, the expressions of *JrMYB44* in walnut [43], five *PgbZIP* genes in ginseng seedlings [44], and *OsbZIP62* in rice [45] were first increased, and then, decreased as the treatment time of PEG6000 was increased. Similarly, the expression levels of *GmNAC3* and *GmNAC4* in soybean roots were first increased, and then, decreased under the treatment of PEG6000 [36,37].

Studies have shown that different TFs generally function at different sites of multiple metabolic pathways. For example, two TF genes of *Arabidopsis thaliana*, *ANAC087* and *ANAC046*, were expressed in large quantities in the roots and involved in apoptosis [46], while *TaRNAC1* was highly expressed in wheat roots to improve the growth of the root system and enhance drought tolerance [47]. Furthermore, studies have shown that the expression levels of NAC TF genes such as *GmNAC3* and *GmNAC4* are generally high in soybean roots [36,37]. However, our results showed that the highest gene expression levels of *GmNAC19* were revealed in leaves during the seedling, flowering, and podding stages. Similarly, the highest expression level of *HaDREBA5* was detected in the leaves of sunflower, with the overexpression of *HaDREBA5* enhancing tolerance to low temperature, drought, and salt in tobacco plants [48].

Moreover, our results for the gene expression of *GmNAC19* evaluated in transgenic soybean hairy roots showed that the gene expression of *GmNAC19* and its expression pattern in the control group were consistent with those derived from the transcriptome sequencing analysis, and the gene expression level of *GmNAC19* in the transgenic group showed an increasing pattern, reaching the highest level at 12 h. These results indicated that *GmNAC19* significantly responded to the treatment of drought stress. Similarly, previous studies showed that under drought stress, multiple genes were expressed in plants to regulate the downstream products to alleviate the detrimental effects caused by drought [13]; both *ANAC087* and *ANAC046* were highly expressed in the roots of *A. thaliana* [46], and *TaRNAC1* was largely expressed in wheat roots [47], to improve the drought tolerance of plants.

### 3.2. Enhanced Drought Tolerance Conferred by Soybean Transcription Factor Gene GmNAC19

Our results revealed enhanced drought tolerance in transgenic *Saccharomyces cerevisiae* INVSc1 with *GmNAC19*, as observed in its improved growth performance based on the number of transgenic yeast cells grown on the YPD medium containing mannitol. These results indicated the alleviating effect of *GmNAC19* on drought stress, which was consistent with the results previously reported, showing the significantly improved drought resistance of yeast induced by the overexpression of walnut *JreIF1A* [49]. Furthermore, our results revealed improvements in seed germination percentage, root growth, drought tolerance, and recovery ability after rehydration in the transgenic *A. thaliana* with *GmNAC19* in comparison to those of WT plants under drought stress either simulated by PEG6000 or caused by water loss, indicating that *GmNAC19* could enhance drought tolerance in *A. thaliana*. These results were consistent with those previously reported, showing that the overexpression of *CaNAC46* and *CarNAC4* improved the drought tolerance of *Arabidopsis* [28,34]. Furthermore, previous studies showed that the plant response to adverse environmental conditions is indicated by maintaining a high seed germination rate because seed germination is generally inhibited by abiotic stresses [50], suggesting that soybean yield and quality could be affected in response to environmental stresses, e.g., drought. Moreover, studies indicated that the up-regulation of the transcription factor MYB68 improved the salt tolerance, the number of seeds per pod, and the weight per 100 seeds of soybean, while high levels of soluble sugar and proline were important indicators in response to environmental stresses [51]. In addition, our results showed that under the drought stress treatment with 20% PEG6000, the chlorophyll content of leaves in the transgenic soybean composite lines with *GmNAC19* was higher than that in the control group. These results were consistent with those previously reported, showing that under drought conditions, high chlorophyll contents were revealed in the leaves of soybean lines with overexpression of *GmNAC3* and *GmNAC4* [36,37], while the overexpression of *MdNAC1* promoted photosynthesis in apple [30], ultimately improving the drought tolerance of the plants.

### 3.3. Variations in Physiological and Biochemical Indices Related to Drought Tolerance Conferred by the Gene GmNAC19

Studies have shown that under drought stress, the level of reactive oxygen species (ROS) harmful to plants is increased [36], while antioxidant enzymes play an important role in reducing the level of ROS and resisting oxidative stress in plants [8,52]. Under drought conditions, the antioxidant and osmotic regulatory systems are generally activated, as indicated by the enhancement in antioxidant oxidase activity and the accumulation of soluble sugar, soluble protein, and proline. Therefore, variations in drought tolerance could be evaluated based on the contents of osmoregulatory substances and the activities of antioxidant enzymes (e.g., SOD, CAT, and POD) involved in the elimination of ROS [6]. Furthermore, studies have revealed the cytotoxic effects of MDA, including the inhibition of gene expression and the promotion of cell death, while under drought stress, plants produce a large quantity of MDA, ultimately causing damage in plants [53]. It is well known that numerous TFs are involved in improved resistance to adverse conditions by increasing the enzymatic activities of SOD, POD, and CAT, increasing the contents of soluble protein, soluble sugar, and proline, and decreasing the content of MDA. For example, previous studies showed that the accumulation of proline in transgenic *Arabidopsis* with *CarNAC4* was increased [34], the contents of proline and soluble sugar in rice with overexpression of *OsNAC066* were increased [35], the content of proline in transgenic *A. thaliana* with *GmWRKY16* was increased [54], the enzymatic activities of SOD, POD, and CAT and the content of proline were increased, and the content of MDA was decreased in transgenic *Arabidopsis* with overexpression of *VvNAC17* [33]. These results were consistent with the findings revealed in our study, showing enhanced drought tolerance, as observed in the increased contents of soluble protein and soluble sugar and the enzymatic activities of SOD, POD, and CAT, and the decreased content of MDA, in transgenic *A. thaliana* with *GmNAC19* treated with drought stress either simulated by PEG6000 or caused by water loss. Furthermore, our results showed that the proline content in the transgenic *A. thaliana* was significantly increased as the treatment time of drought stress was increased, suggesting a higher level of adaptation to drought stress than the control group. These results were consistent with those previously reported, with one study showing an increased concentration of proline in transgenic lemon with *FcWRKY40* [55].

We further evaluated the contents of osmoregulatory substances, antioxidant enzyme activities, and the content of MDA in the hairy roots, stems, and leaves of soybean. Our results revealed increased contents of soluble protein, soluble sugar, and proline, enhanced enzymatic activities of SOD, POD, and CAT, and decreased content of MDA in the transgenic soybean with *GmNAC19* compared to those in the control group. These results were consistent with those based on the transgenic *A. thaliana* with *GmNAC19* and in accordance with those previously reported. For example, increased enzymatic activities of SOD and POD and decreased content of MDA were revealed in birch with overexpression of *BpMYB123* under drought stress [56], while decreased enzymatic activities of SOD, POD, and CAT and increased content of MDA were detected in rice with knockout of *OsNAC006* under drought stress [57]. Moreover, three soybean *NAC* genes were revealed to have largely the same alleviating effect on the enhancement of drought tolerance, i.e., the enzymatic activities of POD and CAT and the content of proline were increased in soybean lines with overexpression of *GmNAC3* and *GmNAC4*, the enzymatic activity of SOD and the content of proline were increased in soybean lines with overexpression of *GmNAC8*, and the enzymatic activity of SOD and the content of proline were decreased in soybean lines with defective *GmNAC8* [36,37,38]. Our results showed that in the hairy roots, stems, and leaves, the enzymatic activity of POD and the chlorophyll content of transgenic soybean were higher than those of the control group, indicating that the transgenic soybean with *GmNAC19* could regulate POD activity and chlorophyll content to maintain the photosynthesis, respiration, material accumulation, and growth and development of plants, ultimately enhancing their drought tolerance. These results were consistent with those previously reported in various crop and model plants [36,37,56]. For example, the overexpression of cherry *ChNAC1* in *Arabidopsis* caused an increase in the contents of chlorophyll, water, proline, and protein, as well as the enzymatic activities of POD and SOD [58]. Furthermore, the overexpression of *ThNAC4* in both *Tamarix* and *Arabidopsis* enhanced the activities of antioxidant enzymes (e.g., SOD and POD) and the contents of osmoprotectants (e.g., proline) under stress conditions [59]. Moreover, the overexpression of *SlNAC6* in tomato caused a significant delay in growth with enhanced tolerance to PEG stress, decreased water loss and oxidative damage, and increased levels of proline content and antioxidant enzyme activity [60].

### 3.4. Proline Metabolism in Transgenic Soybean and Arabidopsis thaliana with GmNAC19

Numerous studies have shown that the accumulation of proline in plants is positively correlated with the improvement in stress resistance [61]. It is well known that proline is accumulated in plant cells to maintain cell turgor [62], improve protein stability and protect membrane integrity by binding with hydrogen bonds [63], and protect cells by increasing water absorption potential and promoting enzyme activation [64]. Proline is also a powerful antioxidant defense molecule, metal-chelating agent, protein stabilizer, ROS scavenger, and inhibitor of apoptosis [62]. However, studies have shown that excessive accumulation of proline could lead to impaired cellular and physiological functions [65]. In our study, the results showed that under drought stress either simulated by PEG6000 or caused by water loss, the contents of proline in *A. thaliana* were revealed to have an increasing pattern as the treatment time of drought stress was increased, while the content of proline in the transgenic *A. thaliana* was lower than that in the control group. These results were consistent with those previously reported, showing that under drought stress, most transgenic ryegrass with *CBF1* was revealed to have lower content of proline than that of the control group [66].

**Figure 17:**
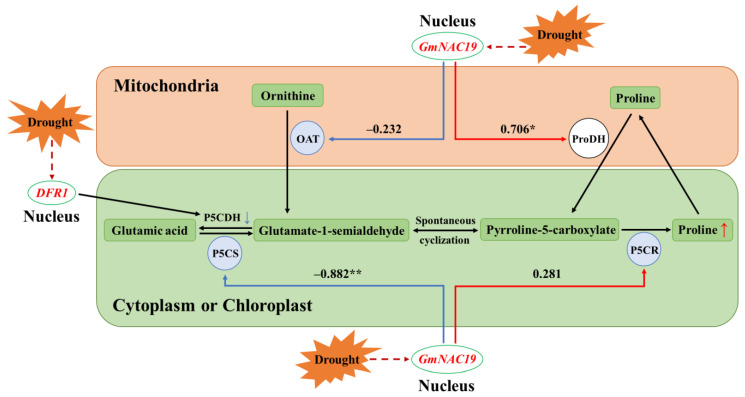
Proline metabolic pathway in soybean. Positive and negative correlations between the gene expression of *GmNAC19* and each of the four genes involved in the proline metabolic pathway (i.e., *P5CS*, *OAT*, and *P5CR* involved in the synthesis of proline and *ProDH* involved in the breakdown of proline) are indicated by blue and red arrowed lines, respectively, with the Pearson correlation coefficients indicated next to the lines (Table 2). Symbols “*” and “**” indicate significant differences in the correlation coefficients determined with *p* < 0.05 and *p* < 0.01, respectively (Table 2). Symbols “↑” and “↓” indicate up-regulation and down-regulation, respectively. OAT, ornithine aminotransferase; P5CS, delta-1-pyrroline5-carboxylate synthase; P5CDH, delta-1-pyrroline-5-carboxylate dehydrogenase; P5CR, pyrroline-5-carboxylate reductase; ProDH, proline dehydrogenase.

In order to further explore the relationships between the gene expression level of *GmNAC19* and the content of proline, the variations in the relative expression levels of four key genes involved in the proline metabolic pathway were evaluated in the transgenic soybean composite lines, i.e., three genes (*P5CS*, *P5CR*, and *OAT*) related to the synthesis of proline and one gene *ProDH* related to the breakdown of proline [67]. The results showed that the contents of proline in the hairy roots, stems, and leaves of the transgenic soybean composite lines were consistently higher than those in the control group. Furthermore, the results of the correlation analysis showed that in the control group, the gene expression level of *GmNAC19* was significantly positively correlated with that of *P5CS*, *OAT*, and *P5CR*, whereas in the transgenic group, the gene expression level of *GmNAC19* was significantly positively and negatively correlated with that of *ProDH* and *P5CS*, respectively (Figure 17). These results indicated that *GmNAC19* was involved in the regulation of proline content by regulating the expression of genes involved in the proline metabolic pathway, ultimately maintaining the dynamic balance between the content of proline and the level of drought stress to improve drought tolerance in soybean. These results were consistent with those previously reported [36,37,68], showing that the overexpression of many TF genes was positively correlated with the expression of key genes involved in the proline metabolic pathway, ultimately increasing the content of proline and adaptation to adverse conditions. For example, the TF gene *DFR1* maintained the functional activities of proline and improved drought tolerance in *Arabidopsis* through the down-regulation of glutamate synthesis based on guanidinosuccinic acid, ultimately promoting the synthesis of proline and drought tolerance [65]. Furthermore, our results showed that under drought stress, both the transgenic *A. thaliana* and soybean composite lines were revealed to have enhanced levels of soluble protein compared with the control group. However, these increases were not comparable with those of soluble sugars, whereas similar variations were observed between the contents of soluble protein and proline, suggesting that proline was not only involved in the regulation of the plant response to drought stress, but also used as a material for the synthesis of soluble protein. These results were consistent with those previously reported, showing that plants responded to adverse conditions by regulating the content of proline, e.g., the synthesis level of proline was decreased in *A. thaliana* with knockout of *P5CS*, while the expression of *P5CS* was up-regulated in rice with overexpression of *OsWRKY50* under salt stress [68]. In summary, the expression levels of these key genes were crucial for the accumulation of proline under drought stress.

## 4. Materials and Methods

### 4.1. Plant and Microbial Materials

Both soybean variety ‘Jiyu47’ and *Arabidopsis thaliana* Colombian (Col-0) WT were planted in a growth chamber with varied temperatures and photoperiod cycles for different experiments (below). Bacterial strains (i.e., competent cells of *Agrobacterium rhizogenes* K599, *A. tumefaciens* AGL0, and *Escherichia coli* DH5α), plasmid pCAMBIA3301-GFP, and yeast expression vector pYES2 were kept in stock at the Crop Germplasm Innovation Laboratory, Jilin Agricultural University. Competent cells of *Saccharomyces cerevisiae* INVSc1 were obtained from Coolaber (Beijing, China), with the cloning vector pMD18-T purchased from Takara Co., Ltd. (Beijing, China).

### 4.2. Cultivation and Treatment of Soybean Materials

To perform the transcriptome sequencing analysis, soybean seeds were sterilized in 2% sodium hypochlorite for 10 min, washed 5 times with sterile water, and sown in a mixture of nutrient soil and vermiculite (3:1; *v*/*v*). The seedlings were transferred to 1 L black plastic pots containing Hogland nutrient solution, which was replaced every 3 d. The 7-day-old soybean plants were selected and treated with 20% PEG6000 (Solarbio Science and Technology Co., Ltd., Beijing, China) to simulate drought stress for 24 h. Root tips of 3 cm in length were collected at 0, 6, 12, 18, and 24 h and stored in liquid nitrogen for subsequent transcriptome sequencing analysis. The raw data of transcriptome sequencing analysis were deposited in the National Center for Biotechnology Information database (https://www.ncbi.nlm.nih.gov/sra/; accessed on 30 October 2023; BioProject accession PRJNA1033409).

To evaluate the gene expression of *GmNAC19*, soybean seeds were sown at the experimental base of Jilin Agricultural University. Different organs (i.e., roots, stems, leaves, flowers, and pods) were collected at three developmental stages (seedling, flowering, and podding), quickly frozen in liquid nitrogen, and stored in a refrigerator at −80 °C for subsequent gene expression analysis of *GmNAC19*.

To establish the transgenic soybean composite lines with *GmNAC19*, soybean seeds were first sterilized, then sown with 1–2 cm intervals in the mixture of nutrient soil and vermiculite (1:1; *v*/*v*), and grown for 6–8 d in the dark. Then, the cotyledon hypocotyls were infected with *Agrobacterium rhizogenes* K599, followed by hydroponic culture with Hogland nutrient solution. At 15–20 d, the primary roots were removed to allow the recovery growth of hairy roots. Soybean plants infected with *A. rhizogenes* K599 without *GmNAC19* were used as the control group and infected with *A. rhizogenes* K599 transformed with *GmNAC19* as the transgenic group. The transgenic soybean composite lines were selected as previously reported [69,70], with slight modifications. The fluorescence exciter (LUYOR-3415RG Hand-Held Blue Green Lamp, LUYOR Instrument Co., Ltd.; Shanghai, China) was first used to verify the successful transformation of soybean hairy roots with soybean *GmNAC19*, i.e., the positive hairy roots were identified by fluorescent light. Then, the identification of positive hairy roots was further confirmed by PCR analysis, i.e., the genomic DNA was extracted and used to PCR-amplify both the 35S promoter and the *bar* gene, and then, the PCR products were evaluated using electrophoresis. The transformation efficiency in this experiment was 93%, i.e., in a total of 50 soybean sprouts infected by transgenic *Agrobacterium rhizogenes* K599 with *GmNAC19*, 45 survived with the development of hairy roots, with a total of 42 positive plants identified. Briefly, the soybean seeds were sown at the same time, and later, the infection by *Agrobacterium rhizogenes* K599 transformed with *GmNAC19* simultaneously using the soybean sprouts of the similar developmental stages. Then, the infected plants were grown at the same time, with fast-growing or slow-growing plants removed and the plants of largely the same developmental stages chosen for further drought stress experiments. The soybean seedlings of the same developmental stages were grown in a nutrient solution containing 20% PEG6000 in a growth chamber (22 °C with relative humidity of 50% and a photoperiod cycle of 12 h dark and 12 h light with light intensity of 360 μmol m^−2^ s^−1^) and sampled at 0, 1, 3, 6, 12, and 24 h. Root tips of 3 cm in length were collected and quickly frozen in liquid nitrogen and stored in a refrigerator at −80 °C for later use.

### 4.3. Cultivation and Treatment of Arabidopsis thaliana

Seeds of *A. thaliana* were first vernalized 4 °C overnight, and then, sown in nutrient pots with the mixture of nutrient soil and vermiculite (1:1; *v*/*v*). The transgenic experiment of *GmNAC19* using the expression vector pCAMBIA3301-GFP was completed via the flower-dipping method using *Agrobacterium tumefaciens* AGL0. The transgenic plants of the T3 generation of *A. thaliana* with *GmNAC19* were used as the experimental group, while the control group contained the non-transgenic plants. The seeds of both WT and transgenic *A. thaliana* plants were evenly sown on the MS medium containing PEG6000 at 0%, 3%, 6%, and 9%, with the seed germination percentage calculated and the root length and diameter measured at 15 d. The root diameter was measured around the thickest place immediately below the junction of the stem and root under an inverted biological microscope (DM IL LED, Leica Microsystems Trading Co., Ltd., Shanghai, China). The phenotypic variations were observed in both the WT and transgenic *A. thaliana* plants with *GmNAC19* treated with 6% PEG6000 for 15 d, and then, rehydrated for 5 d. The 100-seed weights of both the WT and transgenic *A. thaliana* plants with *GmNAC19* were measured based on 100 seeds (*n* = 3) of five lines of both the WT and transgenic plants of the T3 generation using a one ten-thousandth electronic analytical balance (AS 220.X2, Suzhou Peike Laboratory Instrument Technology Co., Ltd., Suzhou, China). Then, the WT and one line (OE-6) of T3 generation transgenic plants of *A. thaliana* were grown on MS medium containing 0% and 6% PEG6000, respectively. Samples were collected at 15 d to determine the physiological and biochemical indices related to drought tolerance, including the contents of soluble protein, soluble sugar, proline, and MDA, and enzymatic activities of SOD, POD, and CAT. Plants at the seedling stage were selected and treated with drought stress induced by water loss for 15 d, with samples collected and stored at 0, 5, 10, and 15 d for the subsequent determination of physiological and biochemical indices. The seedlings were cultured in a growth chamber at 22 °C with a relative humidity of 60% and a photoperiod cycle of 8 h dark and 16 h light (light intensity 310 μmol m^−2^ s^−1^).

### 4.4. Fluorescence-Based Quantitative Real-Time PCR

Total RNA of plant samples was extracted using TIANGEN’s RNAiso Plus reagent (Tiangen Company, Beijing, China). TransScript^®^ Uni All-in-one First-strand cDNA Synthesis SuperMix for qPCR kit (TransGen Biotech Co., Ltd., Beijing, China) was used for both reverse transcription and qRT-PCR experiments using *GmEF1A* as an internal reference gene. Each qPCR experiment was repeated with three biological replicates, with the relative gene expression calculated according to the method of 2^−ΔΔCt^. The primers used in the qPCR experiments were synthesized by Shanghai Biotech Bioengineering Co., Ltd. (Shanghai, China; Table 4).

### 4.5. Validation of Drought Resistance in Saccharomyces cerevisiae

The recombinant plasmid pYES2-*GmNAC19* was transformed into competent cells of *Saccharomyces cerevisiae* INVSc1 (Coolaber, Beijing, China) by following the manufacturer’s instructions. The yeast solution was coated on SD/-Umedium single-deficient medium and cultured in the inverted mode at 29 °C for 2–3 d. Single clones were picked for yeast solution and plasmid PCR detection. Ex-Taq, plasmid extraction, and gel recovery kits were purchased from Takara Co., Ltd. (Dalian, China). Then, the yeast solution was further cultured until the OD_600_ reached 0.5; after the gradient dilution, the yeast solution was sampled and cultured on YPD solid medium containing mannitol of two different concentrations (0.25 and 0.5 M) in the inverted mode in an incubator at 29 °C. DNA sequencing was performed by Shanghai Biotech Bioengineering Co., Ltd. (Shanghai, China).

### 4.6. Determination of Physiological and Biochemical Indicators Related to Drought Tolerance

The content of chlorophyll was measured using a chlorophyll meter SPAD-502 Plus (Konica Minolta, Tokyo, Japan). The content of soluble protein was determined using the Kaumas Brilliant Blue G-250 method. The content of soluble sugar was detected by the anthrone method. Bovine serum albumin (BSA) and glucose of analytical grade were purchased from Solarbio Science and Technology Co., Ltd. (Beijing, China). The content of MDA was determined by the thiobarbituric acid method. The content of proline was measured using the sulfosalicylic acid method. The enzymatic activities of SOD, POD, and CAT were evaluated using the nitrogen blue tetrazolium method, the guaiacol method, and the H_2_O_2_ UV-absorption method [37,71,72], respectively.

### 4.7. Statistical Analysis

Each experiment was performed with three or more biological replicates. SPSS Statistics 26 was used to analyze the data, which were presented as mean ± standard deviation. Statistical significance was determined by Student’s *t* test and either one-way or two-way analysis of variance (ANOVA) followed by a post hoc Tukey’s test. GraphPad Prism 9 was used to generate graphs to present the results of the statistical analyses. Pearson correlation analysis was performed to investigate the relationships between the gene expression of *GmNAC19* and the expressions of 4 key genes (i.e., *P5CS*, *OAT*, *P5CR*, and *ProDH*) involved in proline metabolism in the control and transgenic soybean hairy roots with *GmNAC19*.

## 5. Conclusions

Based on a comprehensive analysis of the gene expression patterns of *GmNAC19* in transgenic soybean composite lines and *Arabidopsis thaliana* under drought stress, our investigations explored the molecular response of the soybean TF gene *GmNAC19* to drought stress either simulated by PEG6000 or caused by water loss. The germination rate of transgenic *A. thaliana* seeds was improved under drought conditions with the promotion of rooting and improved recovery ability after rehydration. The variations in a group of physiological and biochemical indices involved in drought tolerance suggested that the beneficial effects of *GmNAC19* on drought tolerance in both soybean and *Arabidopsis* were achieved by regulating the contents of soluble protein, proline, and soluble sugar, as well as the activities of antioxidant enzymes. However, it is realized that although some of the experiments showed evident effects of this gene on the drought-related biochemical and physiological indices, a stabilized pattern of these effects was not observed due to the lack of an extended period of treatment time. It is also noted that our study of this drought-resistance gene in soybean was performed under a drought condition generated by treatment with PEG6000, and further investigations under actual drought (i.e., reduced water supply over time) are necessary to verify the findings of the drought-resistance effects of this gene in soybean, e.g., the evaluation of the yield and quality of beans as well as soybean plant growth parameters. Our study provided a candidate gene for the molecular breeding and development of drought-tolerant crop plants.

## Figures and Tables

**Table 1:** The 100-seed weight (mg) in both wild-type (WT) and transgenic *Arabidopsis thaliana* with *GmNAC19*. Data are presented as mean ± standard deviation. The statistical difference between the WT and transgenic lines was determined by Student’s *t* test based on *p* < 0.05 (*) and *p* < 0.01 (**).

Transgenic Line	100-Seed Weight	WT Line	100-Seed Weight
OE-1	2.32 ± 0.02 **	WT-3	1.21 ± 0.01
OE-6	3.31 ± 0.03 **	WT-4	1.62 ± 0.02
OE-7	2.62 ± 0.03 *	WT-6	1.61 ± 0.02
OE-9	2.53 ± 0.03 *	WT-7	1.61 ± 0.02
OE-12	2.21 ± 0.02 *	WT-8	1.33 ± 0.01

**Table 2:** Pearson correlation analysis of gene expression of *GmNAC19* with the expressions of 4 key genes (i.e., *P5CS*, *OAT*, *P5CR*, and *ProDH*) involved in proline metabolism in the control and transgenic soybean hairy roots with *GmNAC19* based on the gene expression data presented in Figure 15 and Figure 16, respectively. The control group contains soybean hairy roots transformed with *Agrobacterium rhizogenes* K599 without *GmNAC19*. Symbols “*” and “**” indicate significant differences in the correlation coefficients determined with *p* < 0.05 and *p* < 0.01, respectively.

Group	*P5CS*	*OAT*	*P5CR*	*ProDH*
Control	0.732 *	0.791 *	0.776 *	−0.143
*GmNAC19*-OE	−0.882 **	−0.232	0.281	0.706 *

**Table 3:** Molecular characteristics and responses to drought stress conferred by four *NAC* genes. Symbols “+” and “−” indicate a significant response and no significant response to drought stress, respectively. “n/a” indicates that data are not available. POD, peroxidase; CAT, catalase; SOD, superoxide dismutase; MDA, malondialdehyde.

Gene	Coding Sequence (bp)	Length in Amino Acid	Chromosome	NAM Domain Amino Acid Location	POD Activity	CAT Activity	SOD Activity	MDA Content	Chlorophyll Content
*GmNAC3*	1452	483	n/a	6–132	+	+	+	−	n/a
*GmNAC4*	1650	549	20	20–146	−	+	+	−	n/a
*GmNAC8*	1092	363	n/a	10–139	+	n/a	n/a	n/a	n/a
*GmNAC19*	807	268	13	10–133	+	+	+	−	+

**Table 4:** Primers and their sequences used in qPCR. “-F” and “-R” indicate forward and reverse primers, respectively.

Primer	Sequence (5′→3′)
pYES2-*GmNAC19*-F	CGTTACTAGTGGATCCATGGCCGCAGCAACACA
pYES2-*GmNAC19*-R	AGGGAATATTAAGCTTTCAGAAGGGCCTGGAGAG
*GmNAC19*-3301-F	TCCAGCTCCAGGATCCATGGCCGCAGCAACACA
*GmNAC19*-3301-R	TCAGAAGGGCCTGGAGAGGAGAAAGCTTGGATCC
q*GmNAC19*-F	ATGGCCGCAGCAACACAACT
q*GmNAC19*-R	ATACCACTCTTTCTCTCCGT
q*GmEF1A*-F	TGCAAAGGAGGCTGCTAACT
q*GmEF1A*-R	CAGCATCACCGTTCTTCAAA
q*GmP5CS*-F	TCACTCGCCAAGATGGAAGG
q*GmP5CS*-R	ACTTGCGGCTTCTGAAGGTC
q*GmP5CR*-F	GGGTTCCGTGGAACACTGAT
q*GmP5CR*-R	AGCTCGAAAAGACTGTTATGGC
q*GmProDH*-F	GGTGTCGACAAAGAGGCTG
q*GmProDH*-R	GCGTCTTCCACACCGTACA
q*Gmδ-OAT*-F	AGGGTTTGCAGAGGAAGTAGG
q*Gmδ-OAT*-R	CAGAGGTTCCCTTTGCCTGA

## Data Availability

The raw data of the transcriptome sequencing analysis were deposited in the National Center for Biotechnology Information database (https://www.ncbi.nlm.nih.gov/sra/; accessed on 30 October 2023; BioProject accession PRJNA1033409).

## Supplementary Materials

The following supporting information can be downloaded at: https://www.mdpi.com/article/10.3390/ijms25042396/s1, Figure S1: Drought resistance of wild type (WT) and three lines of transgenic *Arabidopsis thaliana* plants with overexpression of *GmNAC19* (OE-1, OE-6, and OE-7) under normal growth condition (control), drought stress (i.e., treatment of 6% PEG6000), and recovery of drought stress by re-watering, showing the representative images based on three biological replicates.
