# Supplementary material for: Expression Patterns and Molecular Mechanisms Regulating Drought Tolerance of Soybean [Glycine max (L.) Merr.] Conferred by Transcription Factor Gene GmNAC19
Source: Int J Mol Sci. 2024 Feb 18;25(4):2396. doi: 10.3390/ijms25042396 (PMC10889163; doi:10.3390/ijms25042396)
Supplement: Supplementary file 1 [file ijms-25-02396-s001.zip › ijms-2869756-supplementary.pdf]

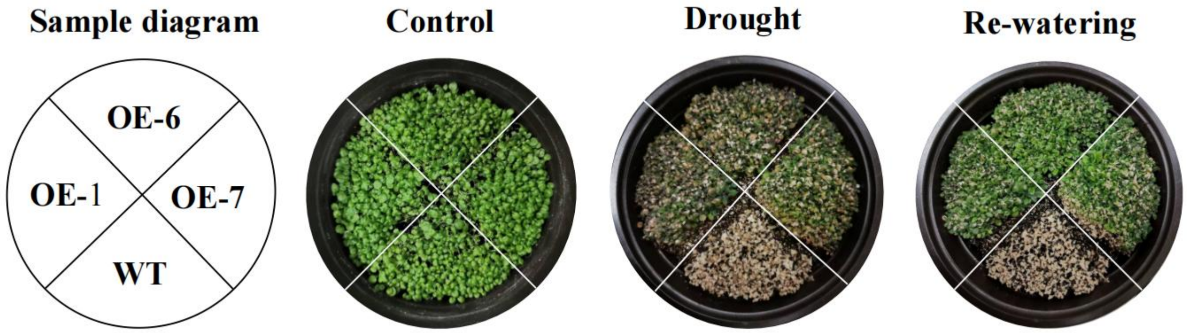

Figure S1. Drought resistance of wild type (WT) and three lines of transgenic *Arabidopsis thaliana* plants with overexpression of *GmNAC19* (OE-1, OE-6, and OE-7) under normal growth condition (control), drought stress (i.e., treatment of 6% PEG6000), and recovery of drought stress by re-watering, showing the representative images based on three biological replicates.
